# Growth and Physiological Responses of Siberian Sturgeon (*Acipenser baerii*) to Partial Fishmeal Replacement by Mealworm and Silkworm Pupae, Individually and in Combination

**DOI:** 10.1155/anu/6272560

**Published:** 2026-01-02

**Authors:** Tahereh Bagheri, Mahmoud Hafezieh, Issa Sharifpour, Homayoun Hossein Zadeh Sahafi, Mansour Sharifian, Shohre Masaeli, Mahmoud Mohseni, Esmaeil Pagheh

**Affiliations:** ^1^ Inland Waters and Aquatics Resources Research Center, Iranian Fisheries Science Research Institute (IFSRI), Agricultural Research, Education and Extension Organization (AREEO), Gorgan, Iran, areo.ir; ^2^ Offshore Fisheries Research Center, Iranian Fisheries Science Research Institute (IFSRI), Agricultural Research, Education and Extension Organization (AREEO), Chabahar, Iran, areo.ir; ^3^ Iranian Fisheries Science Research Institute (IFSRI), Agricultural Research, Education and Extension Organization (AREEO), Tehran, Iran, areo.ir; ^4^ International Sturgeon Research Institute, Agricultural Research, Iranian Fisheries Science Research Institute (IFSRI), Education and Extension Organization (AREEO), Rasht, Iran, areo.ir

**Keywords:** feed utilization, growth, replacement, sturgeon

## Abstract

This study investigated how partially replacing fishmeal (FM) with silkworm pupae (SWPs) (*Bombyx mori*) and yellow mealworm (MW) larvae (*Tenebrio molitor*) affects the growth, health, and metabolism of Siberian sturgeon (*Acipenser baeri*). Four experimental diets were tested: a control with 60% FM and no insect meal, 45% FM with 15% MW, 45% FM with 15% SWPs, and a combined diet containing 45% FM plus both insect proteins: 7.5% MW, as well as 7.5% SWPs (SMW). Fish were reared under controlled conditions, including water temperature 18 ± 1°C, dissolved oxygen 8.5–9.3 mg L^−1^, pH 7.6, and a fixed feeding regime. Sturgeon fed MW and SMW diets showed significantly improved growth, higher final weight, better specific growth rate (SGR), and more efficient protein utilization, along with a lower feed conversion ratio (FCR) compared to the control. Sturgeon fed MW and SMW diets showed significantly improved growth, higher final weight, better SGR, and more efficient protein utilization, along with a lower FCR compared to the control. Digestive enzyme activities (amylase [AMS], protease, lipase [LP]) and key serum metabolites—including protein, albumin, cholesterol, triglycerides, and glucose—were elevated in insect‐fed groups. Immune and antioxidant defenses, such as lysozyme, immunoglobulin M (IgM), glutathione peroxidase (GPX), and superoxide dismutase (SOD), were enhanced, particularly in the SMW and MW treatments, while oxidative stress markers (malondialdehyde [MDA]) were favorably modulated. Gene expression analysis revealed upregulation of growth‐related (growth hormone [GH], IGF1), protein metabolism (target of rapamycin [TOR]), lipid metabolism (Apolipoprotein [ApoE]), and immune genes (interleukin‐1 [IL‐1]), with the strongest response in the combined SMW diet. Overall, partially replacing FM with insect meals, especially the MW and SWP combination, effectively supports growth, metabolism, immune function, and antioxidant capacity in Siberian sturgeon. These findings highlight the promise of insect‐based proteins as a sustainable and effective alternative in Siberian sturgeon aquaculture.

## 1. Introduction

Aquaculture, a key contributor to global food production, is constrained by its reliance on fishmeal (FM) as the primary source of protein and lipids in feeds, which depends heavily on wild fish stocks [1]. FM is widely used in aquafeeds due to its balanced amino acid (AA) profile and high digestibility, which are essential for nutrient absorption, particularly in commonly farmed carnivorous species such as trout, salmon, seabass, and seabream [2]. Contemporary aquaculture practices predominantly depend on marine‐derived ingredients and plant‐based resources to formulate aquafeeds [3]. However, the escalating global demand for aquafeed necessitates the exploration of alternative sources that are both economically viable and environmentally sustainable, to ensure the long‐term productivity and ecological integrity of the industry [4]. Among alternative protein sources, insect‐based feed is gaining recognition as a viable solution [1]. Insects can be cultivated on organic waste materials, thereby supporting circular economy principles and helping to mitigate the environmental footprint of aquaculture practices [5]. Insects offer significant nutritional benefits, characterized by their substantial protein content (ranging from 60% to 80% of their dry weight), considerable fat levels (31%–43%), and a rich profile of essential AAs, vitamins, minerals, and antimicrobial peptides [6]. The acceptance of insect‐derived proteins for aquafeed use has increased following the European Commission’s Regulation (EU) 2017/893, which lifted previous restrictions on processed animal proteins from insects in aquaculture feeds. This regulatory progress underscores the growing global recognition and practical relevance of insect meals as sustainable feed ingredients [7–9]. The selection of insect meals for aquafeeds should consider the nutritional requirements of the target species, including balanced protein and AA profiles, functional nutrients, and support for growth and immunity, while contributing to sustainable resource use [10–13].

The yellow mealworms (MWs) (*Tenebrio molitor*) are well‐known to be cultivated from waste materials and sold as pet feed in live, canned, dried, or powder form [13–15]. Its protein content is high and comparable to FM and soybean meal used in aquafeeds [13]. In vitro evaluation of insect protein hydrolysis revealed similar digestibility of *T. molitor* to FM [8]. Silkworm (*Bombyx mori*) was admitted as the eighth species for inclusion in feed in November 2021 (EU Regulation 2021/1925) [8]. Silkworm pupae (SWPs) are a secondary product of silk manufacturing, as they do not survive the process of silk extraction [16, 17]. Due to their well‐balanced levels of protein and fat, SWPs are suitable for use as animal feed [6].

The use of mixed insect meals, which have different digestion and absorption rates, has not been thoroughly explored in aquafeeds and may offer practical benefits when one insect protein source is limited. Partial replacement of FM with insect meals (up to 15%–30%) has been shown to support growth performance, feed efficiency, and immune responses in Siberian sturgeon (*Acipenser baerii*) [18, 19]. Incorporating two insect species may provide additional benefits by combining nutrients with different digestion rates, potentially enhancing nutrient utilization and health outcomes. Siberian sturgeon (*A. baerii*) stands out for its rapid growth, resilience to stress, and ability to thrive in farming environments, making it one of the most suitable Acipenser species for caviar and meat production [20]. This species consumes insects in its natural habitat [19], suggesting that insect‐based proteins could be a viable component of their diet. Therefore, this study investigates whether partial replacement of FM with MW (*T. molitor*) and SWPs (*B. mori*) can serve as alternative protein sources while enhancing the immunological health of cultured Siberian sturgeon.

## 2. Materials and Methods

### 2.1. Preparation of Trial Diets

*T. molitor* larvae meal (MW) and *B. mori* pupae meal (SWP) were purchased from a local supplier as indicated in the footnote of Table 1. The larvae of *T. molitor* and the pupae of *B. mori* were oven‐dried at 60°C for 48 h and then mechanically ground into full‐fat meal before being utilized in the feeding trials. The proximate composition (crude protein, lipid, ash, and moisture) of these ingredients is shown in Table 1. Crude protein content was determined using the Kjeldahl method, applying a nitrogen‐to‐protein conversion factor (Kp) of 6.25 for FM and 5.60 for insect meals (*T. molitor* and *B. mori*), according to Janssen et al. [21]. Four experimental diets were formulated to be isonitrogenous, isolipidic, and isoenergetic. These diets included: (i) an FM‐based control diet without insect inclusion (FM); (ii, iii, iv) three diets containing 15% insect meal, replacing 15% of FM in the control diet with either MW, SWP, or a combination of 7.5% MW and 7.5% SWP (SMW), respectively. All diets were formulated to meet the nutritional requirements of Siberian sturgeon, based on published literature [20]. Due to the distinct chemical composition of MW and SWP compared with FM, the inclusion of these insect meals necessitated adjustments in other dietary ingredients such as gelatin, starch, and fish oil to maintain nutritional balance across all formulations.

**Table 1:** Experimental diet ingredients and composition for feeding Siberian sturgeon (*A. baerii*) while being cultured at a temperature of 24 ± 2.1°C during a 12‐week study.

Treatments	MW larvae meal	SW pupa meal	FM	MW	SWP	SMW
Ingredients (g kg^−1^)						
Fishmeal (Anchovy Kilka)^a^	–	–	60	45	45	45
Mealworm^b^	–	–	0	15	0	7.5
Silkworm pupa^c^	–	–	0	0	15	7.5
Gelatine	–	–	8	9.5	8	9
Starch	–	–	17	16.5	17	17
Fish oil	–	–	12	11	12	11
Premix^d^	–	–	2.5	2.5	2.5	2.5
VitC	–	–	0.5	0.5	0.5	0.5
Proximate composition (g kg^−1^ as fed)^e^						
DM	89.2	91.7	93.94	92.25	93.52	93.6
CP	48	62	42	42	43	42.5
EE	16.5	13.4	18	18	18.8	17.9
CF	3.4	5.6	0.17	0.48	0.6	0.87
Ash (%)	7.5	8.3	12.6	8.20	7.84	8.15
GE (kcal)	3500	4000	2971	2959	3092	2990

*Note*: MW, 15% mealworm larvae; SMW, 7.5% mealworm larvae + 7.5% silkworm pupa; SWP, 15% silkworm pupa.

Abbreviations: CF, crude fiber; CP, crude protein; DM, dry matter; EE, ether extract; FM, fishmeal; GE, gross energy.

^a^Purchased from Tehran Kilka Powder, Sari, Iran. Proximate composition (g 100 g^−1^, as fed basis): 88.7 DM; 58.8 CP; 10.4 EE; 11.4 ash.

^b^Purchased from Varjavand Hirkan Biotechnology Company, Gorgan, Iran. Proximate composition (g 100 g^−1^, as fed basis): 85.6 DM; 48.5 CP; 15.8 EE; 7.4 ash.

^c^Purchased from Parirou Abrisham Corporation, Amirabad Port, Guilan, Iran. Proximate composition (g 100 g^−1^, as fed basis): 87.5 DM; 62.6 CP; 14.4 EE; 8.1 ash.

^d^Vitamin mixture (IU, µg or mg kg^−1^ diet): DL‐α tocopherol acetate, 45 mg; retinyl acetate, 125,000 IU; DL‐cholecalciferol, 5000 IU; thiamin, 4 mg; riboflavin, 9 mg; pyridoxine, 4.2 mg; B12, 25 mg; folic acid, 2.6 mg; inositol, 1000 mg; biotin, 150 µg; magnesium carbonate, 120 mg; iron sulfate, 20 mg; zinc sulfate,110 mg; copper sulfate, 16 mg; cobalt sulfate, 16 mg; selenium, 0.3 mg.

^e^Values are reported as the mean of duplicate analyses.

The experimental diets were prepared at the aquaculture research facility of the Inland Waters and Aquatic Resources Research Center, Iranian Fisheries Sciences Research Institute, Gharasoo station. Dietary ingredients were selected based on their availability and chemical composition. All dry ingredients were finely ground, accurately weighed, and thoroughly mixed with fish oil. To improve malleability and facilitate pellet formation, 250–500 mL of water per kilogram of feed was gradually added during mixing. The mixture was then processed using a meat grinder fitted with a 5.0 mm die to produce uniform pellets. The pellets were dried at 50°C for 48 h to ensure stability and shelf life [22]. After drying, the diets were stored in sealed dark bags at −20°C until use. The detailed composition of the experimental diets is presented in Table 1.

The fatty acid (FA) and AA profiles of MW larvae, SWPs, and the experimental diets were determined using gas chromatography (GC) and high‐performance liquid chromatography (HPLC), respectively. The FA profiles of MW larvae, SWPs, and the trial diets are presented as g/100 g of total detected FAs (TFAs) in Table 2. The AA composition of MW larvae, SWPs, and the experimental diets is also shown in Table 3.

**Table 2:** Fatty acid composition (g/100 g of total fatty acids) of mealworm larvae, silkworm pupae, and the trial diets.

Fatty acid	MW larvae meal	SW pupa meal	FM	MW	SWP	SMW
C14:0	2.7	0.36	2.41	2.63	1.36	2.58
C14:1	0	0	0	0	0	0.03
C15	0.21	0.61	1.00	0.42	0	0.55
C16	18.81	31.85	19.81	19.69	18.3	18.50
C16:1	1.49	1.40	2.00	2.20	2.28	2.20
C18	3.24	7.57	7.13	5.31	6.55	5
C18:1n9	39.79	35.78	26.5	28.17	31.66	29.37
C18:1n7	0	0	0	0.9	0	0
C18:2n6	26.93	7.82	32.93	29.23	30.25	31.19
C18:3n6	0	0	0	0.54	0	0.38
C18:3n3	1.11	13.96	3.33	4.32	2.91	3.03
C20	0	0	0	0	0	0.39
C20:1	0	0.66	0	0.68	0	0.54
C20:3n6	0	0	0	0	0	0.11
C20:4n6	0.22	0	0.31	0.55	0	0.64
C20:4n3	0	0	0	0	0	0.07
C20:5n3	1.03	0	1.46	1.69	0.88	1.63
C22:4n6	1.37	0	0	0.12	0	0.27
C22:5n3	0	0	0	0.23	0	0.24
C22:6n3	3.65	0	3.13	3.32	5.82	3.37
ΣSFA	24.4	40.4	30.35	28.1	26.2	26.9
ΣMUFA	41.3	37.8	28.5	31.9	33.9	32.1
ΣPUFA	33.3	14.1	41.2	40.0	39.8	40.9
ΣPUFA/ΣMUFA	0.8	0.4	1.4	1.3	1.2	1.3
Σn3	5.8	14.0	7.9	9.6	9.6	8.3
Σn6	28.5	7.8	33.2	30.4	30.3	32.6
Σn3/Σn6	0.2	1.8	0.24	0.3	0.3	0.2

**Table 3:** Amino acid (AA) concentration (% of protein) of mealworm larvae, silkworm pupae, and the trial diets.

Amino acid	MW larvae meal	SW pupa meal	FM	MW	SWP	SMW
Essential AA						
Arginine	3.8	4.1	4.5	4.0	4.2	3.9
Histidine	3.3	3.4	3.4	3.5	3.3	3.4
Isoleucine	5.7	5.6	5.8	5.8	5.8	5.84
Leucine	7.5	7.3	7.6	7.5	7.6	7.6
Lysine	7.2	7.5	6.7	6.8	6.9	6.7
Methionine	2.6	2.4	2.6	2.7	2.6	2.6
Cysteine	0.1	0.2	0.9	0.7	0.8	0.8
Phenylalanine	4.9	5.1	4.3	4.3	4.3	4.3
Tyrosine	5.1	5.4	5.8	5.9	6.0	5.9
Threonine	4.0	4.5	5.3	4.9	5.3	5.2
Valine	6.5	6.3	6.7	6.7	6.8	6.7
Nonessential AA						
Alanine	6.9	6.6	7.1	7.1	6.9	7.7
Aspartic acid	7.5	8.0	7.3	7.1	7.3	7.2
Glycine	5.6	5.3	5.5	5.6	5.5	5.4
Glutamic acid	15.7	15.6	14.3	14.1	14.0	14.2
Proline	7.1	6.8	6.7	6.8	6.7	6.7
Serine	6.6	6.3	6.2	6.4	6.1	6.2

### 2.2. Feeding and Husbandry Practices

A 12‐week growth trial was conducted using 20 circular fiberglass tanks (400 L capacity each) supplied with five replicate tanks assigned to each of the four dietary treatments. Tanks were continuously supplied with artesian well water maintained at a stable temperature of 18 ± 1°C. Weekly monitoring confirmed dissolved oxygen levels ranging from 8.5 to 9.3 mg L^−1^ and a consistent pH of 7.6. Fish were maintained under natural photoperiod conditions from August to October 2024. *A. baerii* were obtained from a commercial sturgeon farm in Kordkouy, Iran, and transported to the experimental facility. Upon arrival, fish were acclimated for 2 weeks under identical rearing conditions. After acclimation, individuals were lightly anesthetized, weighed (initial body weight: 250.46 ± 76.59 g), and randomly distributed into the tanks at a density of ~10 kg m^−3^ (10 fish per tank). Each tank received an equal number of fish. Fish were hand‐fed to apparent satiation three times daily (08:00, 14:00, and 20:00), 6 days per week [23]. To determine actual feed consumption per tank, mortality was monitored, and uneaten feed was collected, dried, and weighed daily. For growth performance assessment, fish from each treatment group were fasted for 24 h, anesthetized, and weighed biweekly. At the end of the trial, final counts and individual growth measurements were recorded. All procedures involving animals were conducted according to the guidelines for the care and use of animals outlined in the National Ethical Framework for Animal Research in Iran and complied with the ARRIVE guidelines [24]. All experimental protocols were approved and conducted following the Ethics Committee of the Iranian Fisheries Science Research Institute (IFSRI; Approval Number 4‐78‐12‐022‐030365).

### 2.3. Sampling Procedures

At the completion of the feeding trial, following a 24 h fasting period, three fish were randomly sampled from each of the five replicate tanks per treatment, resulting in 15 fish per treatment. The sampled fish were anesthetized prior to blood and tissue collection. Blood was drawn from the caudal vasculature using a syringe (~4 mL). The half part was transferred to an EDTA‐containing tube, gently mixed for 2 min, and then refrigerated for subsequent hematological analysis [22]. The remaining blood was centrifuged for 15 min to separate the serum (3000 *g* at 4°C). The resulting supernatant was transferred to a new microtube and then stored at −80°C for later hepatic enzymes and serum biochemical parameters evaluation.

Tissue samples for molecular analysis were collected from the fish target organs, including brain, liver, and intestine. For preparing the samples, after fish were anesthetized, their body surfaces were alcohol‐disinfected, and the target tissues were excised. Tissue samples were immediately placed in liquid nitrogen and kept at −80°C until analyzed genetically. Primers were designed using three software programs information. The gene expression assessment includes (growth hormone [GH], Ghrelin) for growth and appetite, (target of rapamycin [TOR]) for protein metabolism, (Apolipoprotein [ApoE]) for lipid metabolism, and (interleukin‐1β [IL‐1β]) for immunity. Their NCBI accession numbers were presented in Table 4.

**Table 4:** The list of primers used in the experiment of gene expression of *A.baeri* fed trial diets (genes related to protein and lipid metabolism, growth, and immunity).

Primer name	Primer sequence	Accession number	Product size (bp)	Tm (°C)	Efficiency (%)	Application
IGF1‐F	ATCCTCACGCTCTGTACGTG	FJ428828.1	139	60	98	Growth
IGF1‐R	GTTCCTGTTGCCTGTGTTCC					

GH‐F	CTGGTCTCCCCTGAAGAGTC	JX947839.1	181	60	98	Growth
GH‐R	AGAATGCTGACGGGGAGTTT					

Ghrelin ‐F	TGTGAAAAAGCCGGCTGAAC	MG792144.1	129	60	95	Appetite
Ghrelin ‐R	ATCCCGATTTCAAAAGGCGC					

IL‐β ‐F	CTCCTCATCTCCACCTTCATACC	MZ467298.1	116	60	95	Immunity
IL‐β ‐R	AAAGGTGTGTTGGGCGGGC					

TOR ‐F	GCCCAGCTTTCGCATATTGG	XM_034034751.3	95	60	98	Protein metabolism
TOR ‐R	CGCTCGATCTCACCAGAGAC					

ApoE ‐F	CAGCAAGGCAGAGGAGTTCA	GQ851930.1	136	60	96	Lipid metabolism
ApoE ‐R	GGCGTTGAACTGATCCCTCA					

β‐actin‐F	CCTGCGGTATCCATGAGACC	XM_034052523	94	60	94	Housekeeping gene
β‐actin‐R	CCGCCAGACAGTACAGTGTT					

### 2.4. Assessment of Growth Performance

Growth performance parameters were calculated using metrics with the following Equations (1)–(4) and [23]:

(1) Weight gain WG, g= Final body weight g−initial body weight g,

(2) Specific growth rate SGR%∕day=100×ln final body weightg− ln initial body weightg/timedays,

(3) Feed conversion ratio FCR= Total feed supply g, dry matter/tank WG g,

(4) Protein efficiency ratio PER= tank WG g/total protein fed g, DM,

(5) Fulton′s condition factor CF=body weightg/fork lengthcm3×100.

### 2.5. Statistical Analysis

SPSS software (version 9.5.1.733) was provided for data analysis. The Shapiro–Wilk test was considered for assessing the normality of the data, which is more appropriate for datasets with a limited number of replicates. Levene’s test was used to evaluate the homogeneity of variances. One‐way ANOVA followed by Tukey’s post hoc test was then applied to compare the groups. Results are presented as mean ± standard deviation (SD). A statistical expert confirmed that using the Shapiro–Wilk test would not change the outcomes of the analyses.

## 3. Results

### 3.1. Insect and Trial Diets Composition

The proximate composition of the experimental diets was balanced, with DM, CP, and EE contents being generally comparable across formulations. Fiber content increased with insect‐based inclusions (MW, 0.48; SWP, 0.6; SMW, 0.87) compared to the FM diets (0.17), while ash content showed a reduction as insect meal inclusion in trial diets increased. In terms of lipid composition, distinct FA profiles were observed among diets (Table 2). MW larvae meal was characterized by the lowest ΣSFA (24.4%) and the highest ΣMUFA (41.3%), dominated by oleic acid (C18:1n9, 39.8%). By contrast, SWP contained the highest ΣSFA (40.4%), largely due to palmitic acid (C16:0, 31.9%), but the lowest ΣPUFA (14.1%). Trial diets (FM, MW, SWP, SMW) were PUFA‐rich (~40%), with linoleic acid (C18:2n6) as the major component. Among the n3 FAs, SWP meal showed the highest Σn3 content (14.0%), primarily from α‐linolenic acid (C18:3n3, 14.0%), while FM and other trial diets contained moderate levels (7.9%–9.6%). The Σn3/Σn6 ratio was most favorable in the SWP meal (1.8), whereas all other diets displayed a markedly lower balance (0.2–0.3), reflecting their predominance of n6 FAs. AA analysis showed that leucine (7.3–7.6) and isoleucine (5.6–5.84) were relatively stable in all groups, while lysine and tyrosine were higher in SWP meal protein sources and SWP diet. Glutamic acid had the highest amount among nonessential AAs (14.0–15.7), and alanine showed the highest amount in the SMW group (7.7). Other non‐essential AAs showed minor differences between groups. These results indicate that the studied protein sources contain a diverse and appropriate composition of AAs to support growth and performance (Table 3).

### 3.2. Growth Performance

The results showed that the MW group had significantly the highest final weight (967.25 g), specific growth rate (SGR) (1.83%/day), and protein efficiency (2.18), while the FM group showed significantly the lowest weight gain (WG) values (498 g) compared to MW (636.25 g) (see Table 5, *p* ≤ 0.05). Feed conversion ratio (FCR) was 1.1 in MW and SMW groups, with the highest value observed in FM group (1.21). However, it was not statistically significant in treatments except for MW (see Table 5, *p* ≤ 0.05). Condition factor (CF) was not significantly influenced by trial diets, although the lowest value was in SWP group (see Table 5, *p* ≤ 0.05). These results indicate a significant difference in growth performance and feed efficiency between the studied groups.

**Table 5:** The effect of dietary insect meals on the growth performance and feed utilization of Siberian sturgeon (*A. baeri*) fed trial diets (FM, MW, SWP, SMW) over a period of 12‐week study at a temperature of 24 ± 2.1°C.

Growth indices	FM^1^	MW^2^	SWP^3^	SMW^4^
FW	828 ± 80.1^b^	967.25 ± 87.9^a^	848.5 ± 95.2^b^	910.5 ± 54.7^ab^
WG	498 ± 68.1 ^b^	636.25 ± 47.8^a^	510.5 ± 85.7^b^	580.5 ± 73.2^ab^
CF	0.3 ± 0.21^ab^	0.31 ± 0.16^a^	0.25 ± 0.1^b^	0.31 ± 0.2^a^
FCR	1.21 ± 0.34^a^	1.1 ± 0.26^b^	1.13 ± 0.46^b^	1.1 ± 0.38^b^
SGR	1.53 ± 0.8^b^	1.83 ± 0.72^a^	1.5 ± 0.43^b^	1.7 ± 1.2^ab^
PER	1.96 ± 1.2^b^	2.18 ± 1.6^a^	2.04 ± 0.8^ab^	2.11 ± 1.5^ab^

*Note*: Statistically significant difference in Means of each row was shown through different letters (*p* ≤ 0.05). The numbers are derived from three replicates and presented as (mean ± standard deviation). Treatments: ^1^Fishmeal (FM), ^2^Mealworm 15% (MW), ^3^Silkworm 15% (SWP), ^4^Mealworm 7.5% + silkworm pupa 7.5% (SMW).

Abbreviations: CF, condition factor; FCR, food conversion ratio; FW, final weight; PER, protein efficiency rate; SGR, specific growth rate; WG, weight gain.

### 3.3. Enzymatic Activities and Serum Biochemistry

The evaluation of digestive enzymes showed that dietary treatments significantly affected lipase (LP), amylase (AMS), and protease activities (*p* ≤ 0.05) (Figure 1a–c). AMS activity was highest in the MW group (29.5 ± 0.7 U/mg), followed by SWP (24.8 ± 3.1 U/mg), SMW (17.4 ± 2.0 U/mg), and FM (13.7 ± 1.2 U/mg), with MW significantly higher than all other groups (*p* ≤ 0.05) (Figure 1a). LP activity was highest in the SMW group (2.2 ± 0.2 U/mg), followed by SWP (1.6 ± 0.2 U/mg), while FM (0.9 ± 0.1 U/mg) and MW (0.8 ± 0.1 U/mg) exhibited the lowest activities (*p* ≤ 0.05) (Figure 1b). Protease activity was highest in MW (6.7 ± 0.3 U/mg), significantly greater than FM (4.3 ± 0.3 U/mg), while SWP (5.1 ± 0.6 U/mg) and SMW (5.0 ± 0.5 U/mg) were intermediate and not significantly different from MW (Figure 1c).

Figure 1The activities of amylase (AMS) (a), lipase (LP) (b) and total protease (TP) (c) of Siberian sturgeon (*A. baeri*) fed trial diets (fishmeal [FM], mealworm 15% [MW], silkworm pupa 15% [SWP], mealworm 7.5% + silkworm pupa 7.5% [SMW]) over a period of 12‐week study at a temperature of 24 ± 2.1°C. Different letters indicate significant differences (*p* ≤ 0.05).

**Figure:**
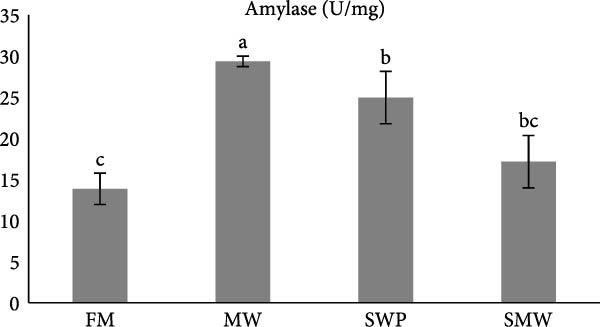
(a)

**Figure:**
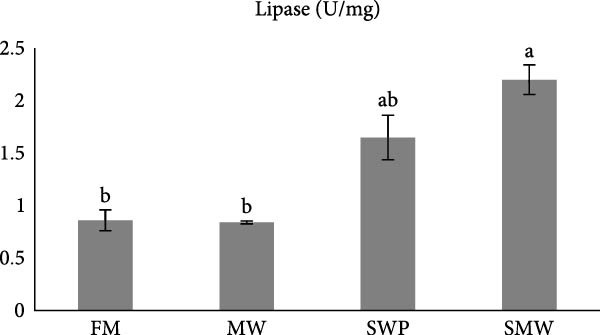
(b)

**Figure:**
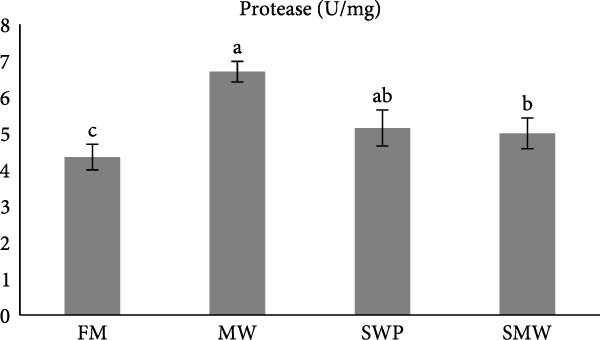
(c)

Hepatic enzymes were also affected by dietary treatments (*p*  < 0.05) (Figure 2a–c). Alkaline phosphatase (ALP) activity differed significantly between MW (246.5 ± 10.6 U/L) and SWP (283.5 ± 7.8 U/L), but no significant differences were observed compared to FM (254.4 ± 10.7 U/L) or SMW (263.4 ± 2.12 U/L) (Figure 2a). Aminotransferase alanine (ALT) levels showed no significant differences among treatments (FM: 22.65 ± 0.64, SWP: 24.55 ± 1.5, MW: 30.4 ± 1.5, SMW: 29.1 ± 0.9 U/L; *p*  > 0.05) (Figure 2b). Similarly, aminotransferase aspartate (AST) activity did not differ significantly between MW (459 ± 4.5 U/L) and SWP (419.5 ± 5 U/L) or between FM (336.5 ± 7.8 U/L) and SMW (354.5 ± 3.5 U/L) (*p*  > 0.05) (Figure 2c).

Figure 2Liver enzyme levels in serum of Siberian sturgeon (*A. baeri*) affected by trial diets (fishmeal [FM], mealworm 15% [MW], silkworm pupa 15% [SWP], mealworm 7.5% + silkworm pupa 7.5% [SMW]) over a period of 12‐week study at a temperature of 24 ± 2.1°C. (a) Alkaline phosphatase (ALP), (b) aminotransferase alanine (ALT), (c) aminotransferase aspartate (AST). Each row with different letters is statistically significant (*p* ≤ 0.05). Different letters indicate significant differences (*p* ≤ 0.05).

**Figure:**
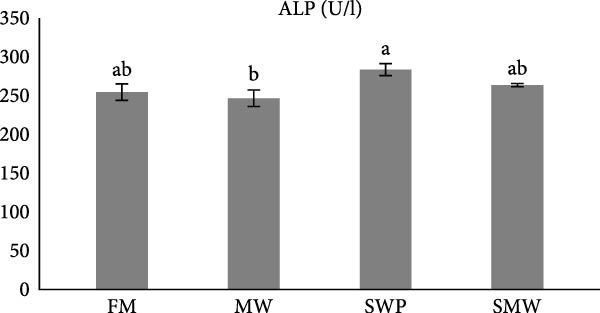
(a)

**Figure:**
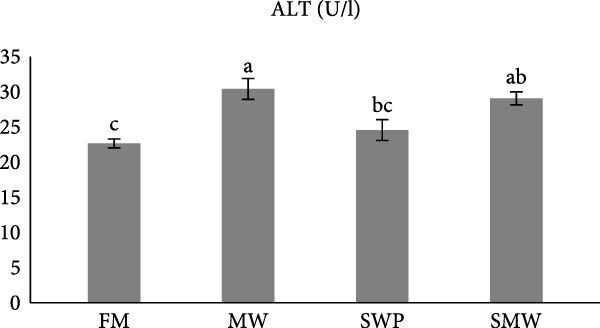
(b)

**Figure:**
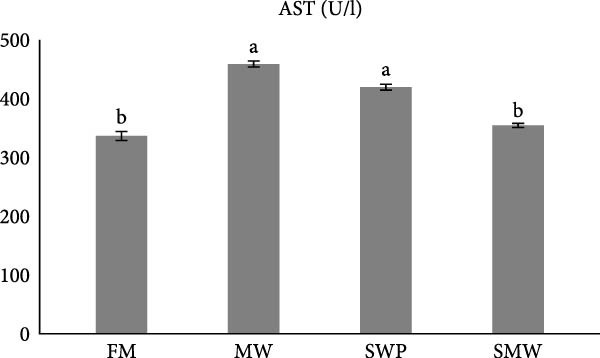
(c)

Key serum metabolites were significantly influenced by trial diets (*p* ≤ 0.05) (Figure 3a–e). Protein levels were highest in SMW (4.66 ± 1.3 g/dL), followed by MW (4.44 ± 0.1 g/dL) and SWP (4.2 ± 0.1 g/dL), with FM showing the lowest concentration (3.88 ± 0.05 g/dL). Albumin was significantly higher in MW (1.715 ± 0.13 g/dL) and SMW (1.605 ± 0.06 g/dL) than in SWP (1.33 ± 0.13 g/dL) and FM (1.22 ± 0.13 g/dL). Cholesterol concentrations were elevated in MW (127.5 ± 7.07 mg/dL), SWP (124 ± 7.07 mg/dL), and SMW (127.5 ± 3.54 mg/dL), while FM showed the lowest value (103.5 ± 2.12 mg/dL). Triglycerides were highest in SMW (390 ± 5.66 mg/dL) and MW (388.5 ± 19.09 mg/dL), followed by SWP (338.5 ± 19.09 mg/dL) and FM (316.5 ± 6.36 mg/dL). Glucose levels were highest in MW (94.4 ± 1.91 mg/dL) and SMW (93.35 ± 3.89 mg/dL), intermediate in SWP (86.05 ± 1.91 mg/dL), and lowest in FM (64.95 ± 3.18 mg/dL). Overall, MW and SMW treatments enhanced serum protein, albumin, and metabolite concentrations, indicating improved metabolic status.

Figure 3The effect of trial diets (fishmeal [FM], mealworm 15% [MW], silkworm pupa 15% [SWP], mealworm 7.5% + silkworm pupa 7.5% [SMW]) on serum metabolites of Siberian sturgeon (*A. baeri*) over a period of 12‐week study at a temperature of 24 ± 2.1°C. (a) Protein, (b) albumin, (c) cholesterol, (d) triglycerides, (e) glucose. Different letters indicate significant differences (*p* ≤ 0.05).

**Figure:**
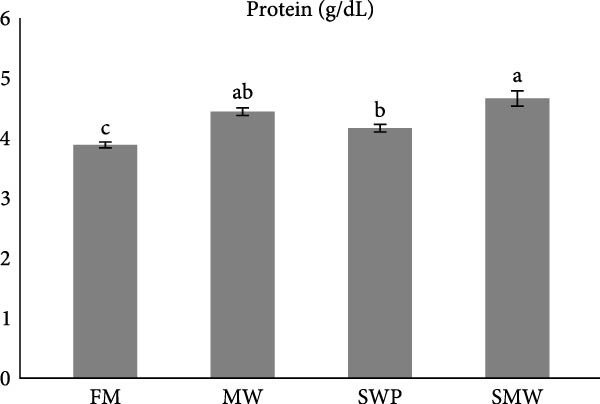
(a)

**Figure:**
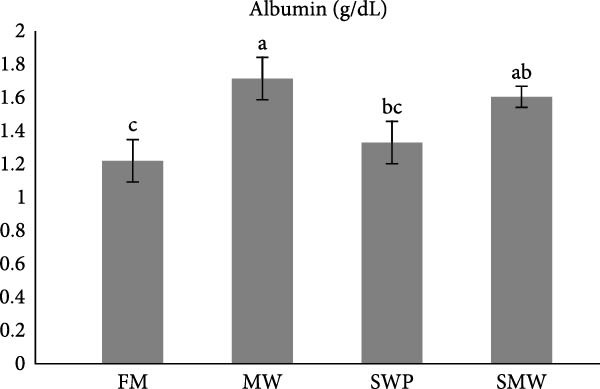
(b)

**Figure:**
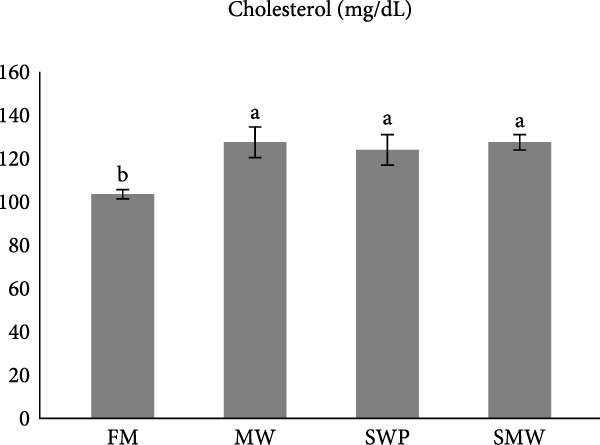
(c)

**Figure:**
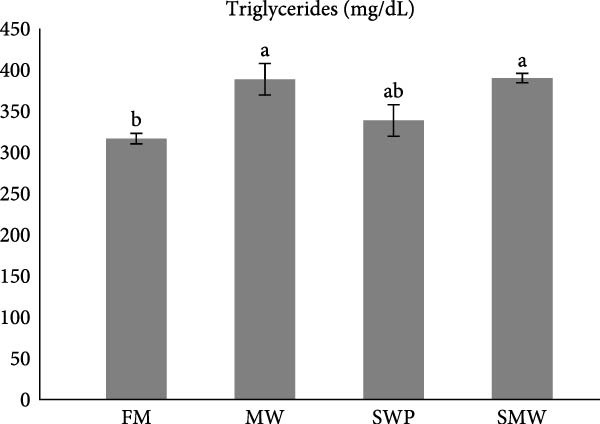
(d)

**Figure:**
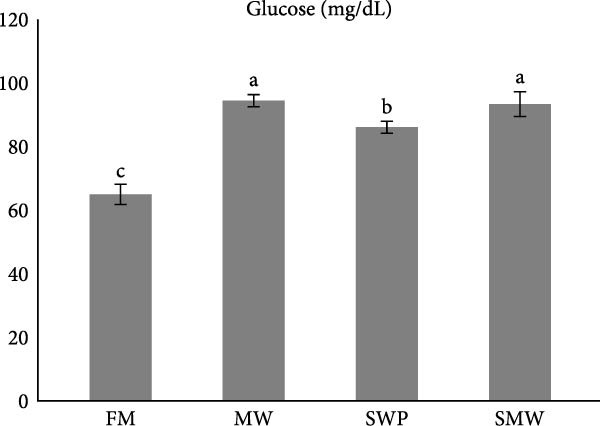
(e)

Innate and humoral immune parameters were significantly affected by dietary treatments (*p* ≤ 0.05) (Figure 4a,b). Lysozyme activity was highest in SMW (36.6 ± 1.27 U/mL/min), followed by MW (34.4 ± 1.27 U/mL/min) and SWP (32.2 ± 1.27 U/mL/min), while FM showed the lowest activity (26.6 ± 1.27 U/mL/min). Immunoglobulin M (IgM) levels followed a similar pattern, being highest in SMW (66.6 ± 1.27 mg/dL), followed by MW (64.4 ± 1.34 mg/dL) and SWP (61.15 ± 1.34 mg/dL), with FM showing the lowest concentration (54.4 ± 1.27 mg/dL). These results indicate that SMW treatment most effectively enhanced both innate and humoral immune responses.

Figure 4The effect of trial diets (fishmeal [FM], mealworm 15% [MW], silkworm pupa 15% [SWP], mealworm 7.5% + silkworm pupa 7.5% [SMW]) on serum immunological responses of Siberian sturgeon (*A. baeri*) over a period of 12‐week study at a temperature of 24 ± 2.1°C. (a) Lysozyme, (b) immunoglobulin (IgM). Different letters indicate significant differences (*p* ≤ 0.05).

**Figure:**
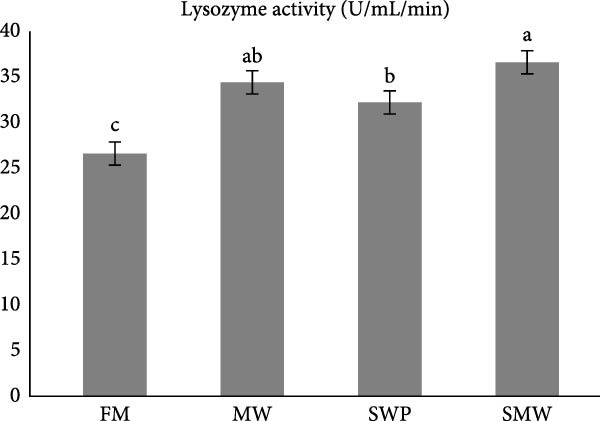
(a)

**Figure:**
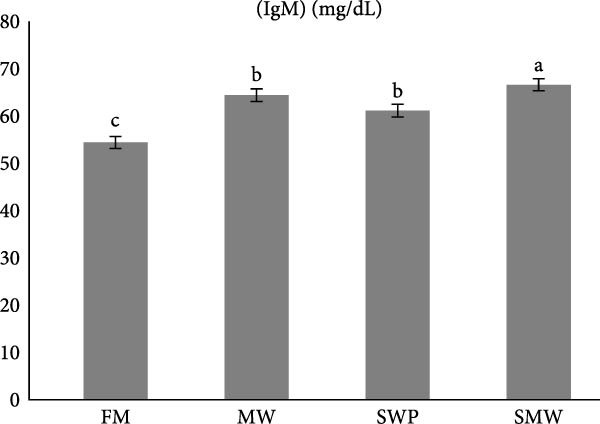
(b)

Antioxidant and oxidative stress parameters also differed significantly among treatments (*p* ≤ 0.05) (Figure 5a–c). Glutathione peroxidase (GPX) activity was highest in SMW (266 ± 12.73 U/mL), followed by MW (222 ± 9.19 U/mL) and SWP (217.5 ± 9.19 U/mL), with the lowest activity in FM (182 ± 12.73 U/mL). Superoxide dismutase (SOD) activity was highest in MW (72.85 ± 3.18 U/mL), followed by SMW (67.7 ± 1.27 U/mL) and SWP (67.15 ± 3.18 U/mL), while FM had the lowest activity (54.95 ± 3.32 U/mL). Malondialdehyde (MDA) concentrations were highest in MW (94.95 ± 0.64 nmol/mL) and SMW (92.75 ± 1.91 nmol/mL), intermediate in FM (84.4 ± 4.45 nmol/mL), and lowest in SWP (75.05 ± 0.64 nmol/mL). These findings indicate that SMW and MW treatments effectively enhanced antioxidant enzyme activities and modulated oxidative stress.

Figure 5The effect of trial diets (fishmeal [FM], mealworm 15% [MW], silkworm pupa 15% [SWP], mealworm 7.5% + silkworm pupa 7.5% [SMW]) on serum antioxidant responses of Siberian sturgeon (*A. baeri*) over a period of 12‐week study at a temperature of 24 ± 2.1°C. (a) Glutathion peroxidase (GPX), (b) malondialdehyde (MDA), (c) superoxide dismutase (SOD). Different letters indicate significant differences (*p* ≤ 0.05).

**Figure:**
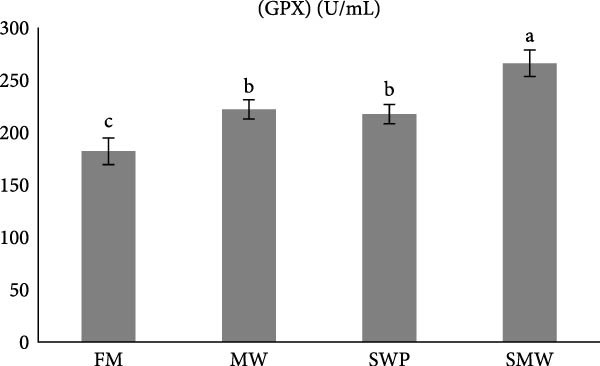
(a)

**Figure:**
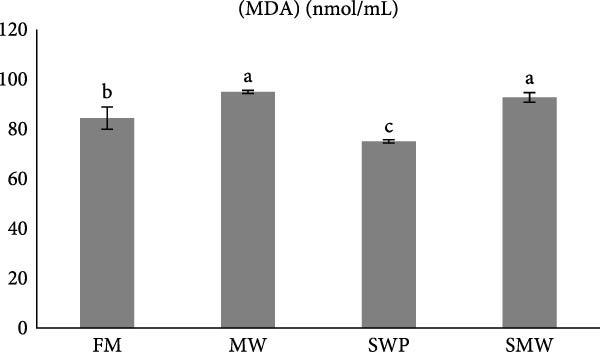
(b)

**Figure:**
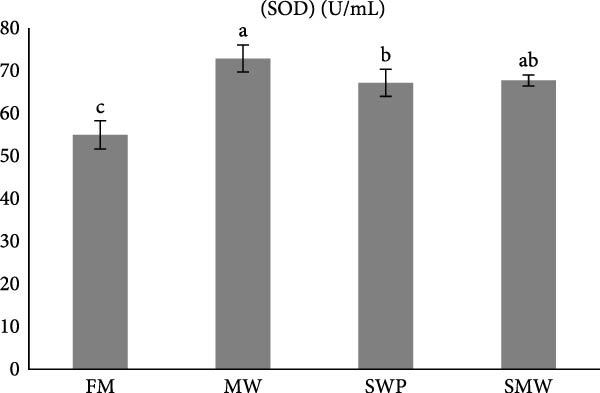
(c)

### 3.4. Assessment of Growth, Metabolic, and Immune Gene Expression

**Figure 6:**
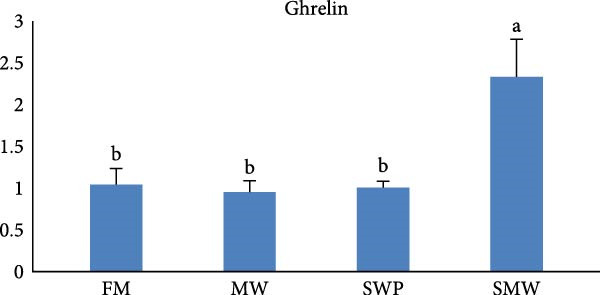
Relative expression of the intestinal ghrelin gene in Siberian sturgeon (*A. baerii*) after 12 weeks of feeding with different trial diets (fishmeal [FM], mealworm 15% [MW], silkworm pupa 15% [SWP], mealworm 7.5% + silkworm pupa 7.5% [SMW]) at 24 ± 2.1°C. Data are expressed as mean ± S.D. (*n* = 4). Bars with different letters denote significant differences (*p* ≤ 0.05).

**Figure 7:**
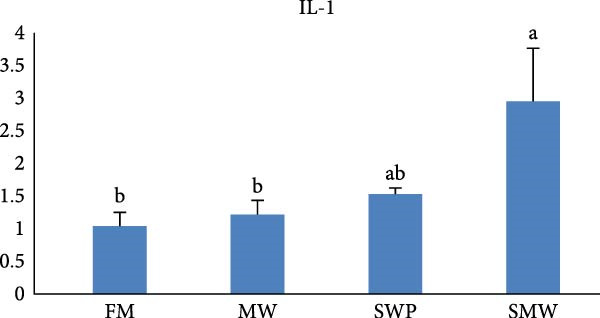
Relative expression of the intestinal interleukin (IL‐1) gene in Siberian sturgeon (*A. baerii*) after 12 weeks of feeding with different trial diets (fishmeal [FM], mealworm 15% [MW], silkworm pupa 15% [SWP], mealworm 7.5% + silkworm pupa 7.5% [SMW]) at 24 ± 2.1°C. Data are expressed as mean ± S.D. (*n* = 4). Bars with different letters denote significant differences (*p* ≤ 0.05).

**Figure 8:**
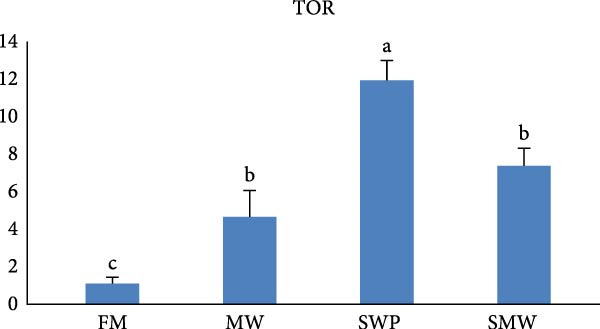
Relative expression of the liver target of rapamycin (TOR) gene in Siberian sturgeon (*A. baerii*) after 12 weeks of feeding with different trial diets (fishmeal [FM], mealworm 15% [MW], silkworm pupa 15% [SWP], mealworm 7.5% + silkworm pupa 7.5% [SMW]) at 24 ± 2.1°C. Data are expressed as mean ± S.D. (*n* = 4). Bars with different letters denote significant differences (*p* ≤ 0.05).

**Figure 9:**
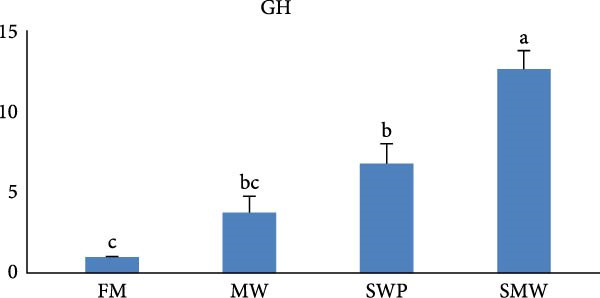
Relative expression of the brain growth hormone (GH) gene in Siberian sturgeon (*A. baerii*) after 12 weeks of feeding with different trial diets (fishmeal [FM], mealworm 15% [MW], silkworm pupa 15% [SWP], mealworm 7.5% + silkworm pupa 7.5% [SMW]) at 24 ± 2.1°C. Data are expressed as mean ± S.D. (*n* = 4). Bars with different letters denote significant differences (*p* ≤ 0.05).

**Figure 10:**
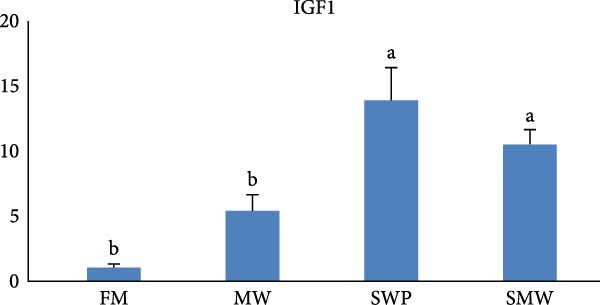
Relative expression of liver insulin‐like factor (IGF1) gene in Siberian sturgeon (*A. baerii*) after 12 weeks of feeding with different trial diets (fishmeal [FM], mealworm 15% [MW], silkworm pupa 15% [SWP], mealworm 7.5% + silkworm pupa 7.5% [SMW]) at 24 ± 2.1°C. Data are expressed as mean ± S.D. (*n* = 4). Bars with different letters denote significant differences (*p* ≤ 0.05).

**Figure 11:**
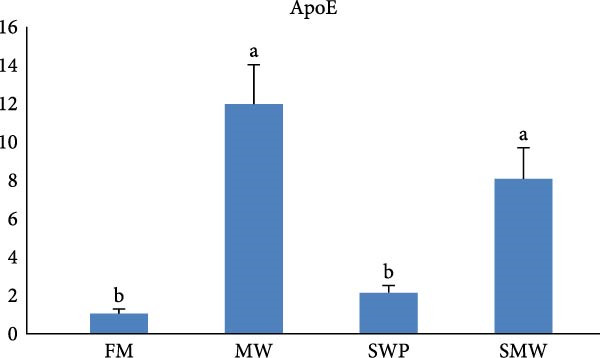
Relative expression of liver Apolipoprotein (ApoE) gene in Siberian sturgeon (*A. baerii*) after 12 weeks of feeding with different trial diets (fishmeal [FM], mealworm 15% [MW], silkworm pupa 15% [SWP], mealworm 7.5% + silkworm pupa 7.5% [SMW]) at 24 ± 2.1°C. Data are expressed as mean ± S.D. (*n* = 4). Bars with different letters denote significant differences (*p* ≤ 0.05).

The intestinal ghrelin gene expression was significantly upregulated under the combined insect‐based diet (SMW; Figure 6, *p* ≤ 0.05). Similarly, intestinal expression of the pro‐inflammatory cytokine gene IL‐1 was significantly upregulated by insect‐based diets (MW and SWP), with the most pronounced increase observed under the combined insect‐based diet (SMW) (Figure 7, *p* ≤ 0.05). Furthermore, genes involved in protein homeostasis, TOR, were significantly upregulated in response to two insect meal inclusion diets, whether separately (MW, SWP) or in combination (SMW), with the highest expression levels observed in the SMW group (Figure 8, *p* ≤ 0.05). Additionally, GH gene expression in brain tissue increased with dietary insect meal inclusion of MW and SWP, with combined insect meal group (SMW) exhibiting significantly greater expression compared to the other treatments (Figure 9, *p* ≤ 0.05). In contrast, insulin‐like factor (IGF1) gene in liver of SWP group significantly increased, followed by combined insect meal group (SMW) (Figure 10, *p* ≤ 0.05).The highest expression of ApoE gene appeared in liver of MW group, followed by combined insect meal group (SMW) (Figure 11, *p* ≤ 0.05). The least gene expression was revealed in FM group for all studied genes.

## 4. Discussion

The growth data analysis revealed that FW, WG, SGR, and PER were significantly enhanced in the MW trial diet containing 15% MW. Moreover, FCR significantly improved in the insect‐based groups, suggesting that fish‐fed insect diets had a better ability to convert food into muscle and increase diet efficiency. However, CF was not significantly affected by insect‐based trial diets (MW, SWP, SMW) compared to FM. It was significantly suppressed by the SWP diet, but no differences were observed among MW, SMW, and FM. In the present study, Fulton’s CF values averaged between 0.25 and 0.26, which are markedly lower than those typically observed in many teleost species but fall in line with patterns reported for sturgeon species [23, 25, 26]. In Siberian sturgeon studies, *K* values < 1 are common and generally attributed to the species’ slender, elongated morphology rather than poor health [23]. It is worth noting that the test diets contained 45% FM and 15% insect meal, without additional growth promoters or AAs. Therefore, the FM component in the test diets likely supplied sufficient levels of essential nutrients that may be lower in insect meals alone. This could partially explain the observed improvements in growth performance and feed efficiency in the insect‐fed groups, highlighting the importance of maintaining a balanced nutrient profile when incorporating alternative protein sources in Siberian sturgeon diets [18, 27]. For instance, in growth trials on Siberian sturgeon using blends of rendered animal proteins to replace FM, *K* values around 0.7–0.8 have been documented without evidence of adverse health effects [26]. Moreover, in our study, the high survival rate and absence of signs of physiological stress further support the interpretation that these low K values are morphological rather than symptomatic of nutritional inadequacy. Concordant with our findings, Józefiak et al. [19] reported that replacing 15% of FM with full‐fat *Hermetia illucens* or *T. molitor* meals did not negatively affect growth performance or survival in juvenile Siberian sturgeon. Similarly, more recent work on black soldier fly larvae (BSFL) meal as a partial replacement showed that incorporation levels up to 30% can enhance body WG, SGR, and feed and protein conversion ratios in Siberian sturgeon (with no detrimental effects on digestibility) [28]. These results lend credence to our observations that insect‐based diets (MW, SWP, SMW) yielded improvements in FCR, WG, SGR, and PER relative to the FM control, without compromising fish health indicators. The fact that we observed no significant differences among dietary treatments in morphometric indices further aligns with previous literature in other fish species fed insect meals (e.g., 29), indicating that inclusion of insect protein often does not alter body shape or condition metrics when diets are properly balanced. Taken together, our results suggest that for Siberian sturgeon, partially inclusion of insect‐derived protein sources (MW) could efficiently replace FM without detriment to health status, and potentially with advantages in feed efficiency and growth performance—provided that AA balance, lipid composition, and overall diet formulation are carefully optimized. In our study, the replacement rate was not too high to render the chitin content of the diets intolerable for fish growth (up to 15%).

Aquaculturists suggest that the inclusion rate of insect meal should be adjusted for each individual species [19]. The inclusion of a novel feed ingredient in animal feeds should be evaluated not only in terms of growth performance but also with respect to its potential effects on health [29, 30]. Indeed, imbalanced dietary formulation can exert detrimental effects on fish physiology, manifested as reduced growth performance, immunosuppression, and metabolic dysregulation [31]. One of the beneficial tools for understanding nutrient utilization of aquaculture species is digestive enzymes evaluation [32]. In sturgeons, which are ancient fishes with unique gastrointestinal physiology and relatively slow growth, the efficiency of nutrient hydrolysis and absorption is an essential determinant of feed performance [33]. Digestive enzyme activities reflect the fish’s capacity to break down macronutrients into absorbable forms and thereby serve as proxies for digestibility and potential growth outcomes [34]. Given the growing interest in sustainable alternatives to FM, such as insect‐derived proteins, measuring enzyme activities in Siberian sturgeon provides direct insight into how novel ingredients influence digestive physiology and nutrient availability. Dietary treatments significantly affected AMS, LP, and protease activities in Siberian sturgeon after 8 weeks of feeding. AMS activity was highest in fish fed the MW diet, significantly greater than the SWPs, mixed (SMW), and FM groups. This suggests that MW inclusion stimulated carbohydrate hydrolysis despite the generally low starch content of insect meals. One possible explanation is the presence of chitin in MW cuticle, which may act as a nonstarch polysaccharide stimulus for carbohydrase secretion [35]. Comparable responses have been reported in other fish fed insect‐based diets, where changes in carbohydrase activity reflect digestive adaptation to structural polysaccharides [36]. The high AMS activity in MW‐fed fish may therefore indicate improved capacity to utilize available carbohydrates and adapt to alternative feed substrates. LP activity was greatest in the SMW group, followed by SWP, while FM and MW diets exhibited the lowest values. This pattern is consistent with the lipid‐rich composition of SWPs, which contain elevated levels of essential FAs compared to FM [37]. The particularly strong LP response in the SMW group suggests a synergistic effect when MW and SWP are combined, potentially reflecting broader lipid substrate availability. Similar LP upregulation has been reported in teleost fed insect meals, where changes in dietary lipid profile induced enhanced lipolytic activity [38]. Elevated LP activity in SWP and SMW groups may therefore predict greater efficiency of lipid hydrolysis and absorption. Protease activity was significantly highest in MW‐fed sturgeon, surpassing the FM group, while SWP and SMW were intermediate. This finding indicates that MW proteins are effectively hydrolyzed by sturgeon proteases, highlighting their potential as a suitable protein source. Previous research has shown that protease activity correlates with protein digestibility in *A. baeri*, *A. naccarii*, as well as *Oncorhynchus mykiss* [33, 34], and similar enhancements of protease activity have been reported in other fish species when insect proteins replaced FM [39]. The elevated protease activity in the MW group suggests favorable adaptation to insect‐derived protein and the potential for efficient AA utilization. Overall, the enzyme activity profiles demonstrate that insect meals elicit distinct digestive adaptations in Siberian sturgeon. MW promoted strong proteolytic and amylolytic responses, SWP stimulated lipolytic adaptation, and SMW produced a synergistic effect on lipid digestion. These findings reinforce the value of digestive enzyme assays as indicators of hydrolytic potential and support the inclusion of insect meals, particularly MW and SWP, as promising FM alternatives in sturgeon diets. Nevertheless, because enzyme assays measure digestive capacity rather than realized absorption, complementary approaches such as apparent digestibility trials or intestinal transporter expression analyses are recommended to confirm nutrient utilization outcomes.

The present study also demonstrates that dietary inclusion of MW, SWPs, and their combination (SMW) significantly modulated hepatic function in Siberian sturgeon (*A. baerii*), highlighting the physiological impacts of insect‐based diets. Hepatic enzyme activities, including ALP, alanine aminotransferase (ALT), and aspartate aminotransferase (AST), are sensitive indicators of liver function and metabolic adaptation to dietary composition [40] as well as liver impairment reflectors [41]. In this study, ALP activity differed significantly between MW and SWP, whereas ALT and AST activities did not vary significantly among treatments. The absence of significant ALT and AST alterations suggests that insect meal inclusion did not induce hepatocellular damage, aligning with previous reports in teleost, where moderate replacement of FM with insect meals maintained normal liver enzyme profiles [42, 43]. Slight ALP differences may reflect minor adjustments in hepatic metabolism associated with lipid and protein processing from insect‐based diets, rather than liver pathology.

Key serum metabolites—including total protein, albumin, cholesterol, triglycerides, and glucose—were enhanced in MW and SMW treatments. The highest protein and albumin levels observed in SMW and MW groups indicate improved nitrogen retention and hepatic protein synthesis, which are linked to growth performance and overall metabolic status [22, 23]. Elevated cholesterol and triglycerides in MW and SMW diets likely reflect the lipid‐rich nature of insect meals, especially SWPs, contributing essential FAs for energy and cellular functions [1]. Increased glucose levels in MW and SMW‐fed sturgeon suggest enhanced carbohydrate utilization and energy availability, consistent with prior observations that insect‐based diets can modulate serum metabolites favorably [36].

The present study demonstrates that insect inclusion, particularly in combination, significantly influenced both innate and humoral immune parameters, as well as antioxidant and oxidative stress responses in Siberian sturgeon. Specifically, the SMW diet promoted the highest lysozyme activity, IgM levels, and GPX activity, while MW also enhanced SOD activity and modulated MDA concentrations, a marker of lipid peroxidation, reflecting improved immune defense and antioxidant capacity. Lysozyme activity and IgM levels were also highest in SMW, followed by MW and SWP, with FM showing the lowest values. The immunostimulatory effects of insect meals have been attributed to bioactive components such as chitin, antimicrobial peptides, and FAs, which can enhance leukocyte activity and Ig production [44, 45]. Indeed, insect‐based diets contain antibacterial compounds as well as chitin, which made it as an antioxidant [46]. The results of antioxidant enzymes and upregulation of IL1 expression indicate that since MW and SWP possess this polysaccharide in its exoskeleton and is expected to enhance the oxidative defense system, potentially mitigating reactive oxygen species generated during metabolism [43]. The observed pattern suggests that SMW‐fed sturgeon provide the most balanced enhancement of antioxidant capacity, likely due to a synergism effect of bioactive compounds from both insects. These findings align with the review by Martínez‐Álvarez et al. [47], who emphasized that antioxidant defenses in fish are shaped not only by abiotic conditions but also by biotic factors such as diet and nutritional composition. The enhanced enzyme activities and modulation of oxidative stress observed in our study suggest that dietary inclusion of SMW and MW supports redox homeostasis and strengthens immune competence, thereby mitigating oxidative damage. In line with previous evidence, these results reinforce the role of targeted nutritional strategies in optimizing fish health through the interconnected regulation of antioxidant systems and immune responses [47]. On the other hand, insect meals, such as MW and SWPs, SWP contain immunomodulatory peptides involved in the regulation of immune response [48, 49]. A novel immunomodulatory peptide purified from the SWP protein hydrolysate significantly upregulated the expression of IL‐1β, suggesting that this compound may play a key role in activating pro‐inflammatory signaling pathways and enhancing the immune response in fish [49]. Additionally, SWP oil has demonstrated notable antibacterial activity [49]. It is also a valuable source of chitin and chitosan, biopolymers known for their pronounced antioxidant and antimicrobial properties [50]. Interestingly, the antibacterial and antifungal activities of chitosan derived from SWP have been reported to be more potent than those of commercially available chitosan [51]. Furthermore, insect meals are naturally richer in saturated FAs (SFAs) compared to conventional FM. The elevated SFA content contributes to enhanced oxidative stability, as SFAs lack double bonds and are therefore less susceptible to lipid peroxidation, ultimately reducing the generation of reactive oxygen species [52]. As a result, diets incorporating insect meals—particularly the combined SMW diet—exhibit lower susceptibility to oxidation, thereby preserving nutritional quality and promoting fish health.

The present study observed a significant upregulation of intestinal ghrelin gene expression in fish fed the combined insect‐based diet (SMW), indicating a pronounced dietary influence on appetite‐regulating and metabolic pathways. Ghrelin is primarily produced in the stomach and intestines of fish, and is a key orexigenic hormone that plays a central role in stimulating feed intake, enhancing nutrient absorption, and coordinating energy balance [10, 53]. The highest ghrelin expression levels, suggesting that insect‐based diets may activate ghrelin‐mediated pathways, thereby improving metabolic efficiency and physiological status in fish. These findings align with previous research demonstrating that dietary components can modulate ghrelin expression in fish. For instance, a study by Zarantoniello et al. [54] reported a dose‐dependent increase in ghrelin gene expression in zebrafish fed diets containing varying levels of BSF prepupae meal. Additionally, Zhang et al. [55] observed that dietary lipid concentrations influenced ghrelin expression in fish, highlighting the role of diet in regulating ghrelin‐mediated pathways. The upregulation of ghrelin expression in response to insect‐based diets may also be associated with the activation of anabolic pathways such as the TOR and GH signaling, as observed in our study. Ghrelin has been shown to interact with these pathways to promote protein synthesis and growth [56]. Furthermore, the modulation of ghrelin expression could influence immune responses, as ghrelin has been implicated in regulating inflammation and immune cell function [56].

Insect meal inclusion, particularly the combined SMW diet, significantly influenced the expression of genes involved in growth, metabolism, and immune regulation in Siberian sturgeon. The pro‐inflammatory cytokine IL‐1 was upregulated in the intestine, especially in the SMW group, indicating a controlled activation of local immune responses. Our study demonstrates that insect meal inclusion, particularly the combined SMW diet, significantly modulated the expression of genes involved in growth, protein metabolism, and lipid homeostasis in Siberian sturgeon. The upregulation of TOR in all insect diets, especially SMW, indicates enhanced protein synthesis and anabolic activity. This finding is consistent with previous studies showing that insect protein inclusion can stimulate TOR signaling and improve protein metabolism in fish [57]. Similarly, IGF1 expression in the liver was significantly elevated in SWP and SMW groups, suggesting systemic growth promotion. This observation aligns with studies reporting that nutrient‐rich insect diets can upregulate IGF1, enhancing growth‐related pathways [54]. Furthermore, the increase in ApoE expression in MW and SMW groups indicates improved lipid transport and energy metabolism, supporting metabolic efficiency. This effect has been documented in studies where insect meals provided bioactive lipids and AAs that favorably modulate lipid metabolism [58]. Collectively, these results demonstrate that insect meal inclusion, especially in combination (SMW), provides bioactive nutrients that synergistically enhance growth, protein metabolism, and immune competence. FM‐fed groups consistently showed the lowest expression of these genes, highlighting the advantage of insect‐based proteins over traditional protein sources in optimizing molecular pathways critical for Siberian sturgeon performance [59].

Siberian sturgeon (*A. baerii*) needs high protein diets [20] and dependency on traditionally FM protein raises concerns on sustainability and cost. Plant protein, such as soybean meal, often has fixed nutrient profiles, limited AA balance, and potential anti‐nutritional factors, which may restrict growth, immune response, and overall health. Insect meals as novel Alternative protein sources offer promising achievements. Future research should focus on developing sustainable aquaculture practices for the cultivation of sturgeon, a species in high market demand.

## 5. Conclusion

Partially replacing FM with MW, SWPs, or their combination (SMW) significantly improved growth, feed efficiency, and protein utilization in Siberian sturgeon, without affecting health or body shape. Insect‐based diets enhanced digestive enzyme activities, supported liver and serum metabolic functions, and strengthened both immune and antioxidant defenses, with the combined SMW diet providing the most balanced benefits. Gene expression analyses confirmed activation of key growth, metabolic, and immune pathways, reflecting the physiological improvements observed. Overall, these results highlight insect meals as a sustainable and effective alternative to FM, and careful diet formulation can further optimize growth, health, and metabolic performance in sturgeon aquaculture.

## Ethics Statement

All procedures involving animals were conducted according to the guidelines for the care and use of animals outlined in the National Ethical Framework for Animal Research in Iran. All experimental protocols were approved and conducted following the Ethics Committee of the Iranian Fisheries Science Research Institute (IFSRI; Approval Number 4‐78‐12‐022‐030365).”

## Disclosure

All authors have read and agreed to the published version of the manuscript.

## Conflicts of Interest

The authors declare no conflicts of interest.

## Author Contributions

Conceptualization was done by Tahereh Bagheri, Issa Sharifpour, and Homayoun Hossein Zadeh Sahafi. Methodology was done by Tahereh Bagheri, Esmaeil Pagheh, and Mahmoud Hafezieh. Software‐related task was done by Tahereh Bagheri. Validation was performed by Mansour Sharifian, Shohre Masaeli, and Mahmoud Mohseni. Formal analysis was performed by Tahereh Bagheri and Issa Sharifpour. Writing – original draft preparation was done by Tahereh Bagheri. Writing – review and editing was done by Tahereh Bagheri. Supervision was done by Homayoun Hossein Zadeh Sahafi.

## Funding

This work is based upon research funded by the Iran National Science Foundation (INSF) under Project Number 4027280.

## Data Availability

Data will be available upon request from the authors.
